# Changes of Serum Adiponectin and Glycated Albumin Levels in Gestational Diabetes Mellitus Patients and Their Relationship with Insulin Resistance

**DOI:** 10.18502/ijph.v49i7.3578

**Published:** 2020-07

**Authors:** Qingju WANG, Juan DU, Fenglian LIU

**Affiliations:** Department of General Medicine, Jinan City People's Hospital, Jinan, China

**Keywords:** Gestational diabetes mellitus, Serum adiponectin, Insulin resistance

## Abstract

**Background:**

We aimed to investigate the changes of serum adiponectin and glycated albumin (GA) levels in gestational diabetes mellitus patients and their relationship with insulin resistance.

**Methods:**

Overall, 137 pregnant women were enrolled from Jinan City People's Hospital, Laiwu District, China from Jan 2015 to Jun 2018. Among them, 71 pregnant women with gestational diabetes mellitus were examined as diabetes group, and 66 normal pregnant women as normal pregnant women group. In addition, 58 normal non-pregnant women of childbearing age who were examined in our hospital during the same period were selected as a control group. The serum adiponectin and GA levels of the three groups were compared, and the relationship between serum adiponectin, GA levels and insulin resistance was analyzed.

**Results:**

The serum adiponectin level of pregnant women in gestational diabetes mellitus (GDM) group was significantly lower than that of normal pregnant women and control group (*P*=0.031, *P*=0.027). The serum GA level of pregnant women in GDM group was significantly higher than that of normal pregnant women and control group (*P*<0.001). Pearson correlation analysis showed that GA was positively correlated with Fasting plasma glucose (FPG), Fasting insulin (FINS) and Insulin resistance index(HOMA-IR) levels (*P*<0.001), while adiponectin was negatively correlated with FPG FINS and HOMA-IR levels (*P*<0.001).

**Conclusion:**

Abnormal levels of serum GA and adiponectin are closely related to insulin resistance in patients with gestational diabetes mellitus. Detection of serum GA and adiponectin levels can diagnose gestational diabetes mellitus quickly and effectively.

## Introduction

Pregnancy in patients with pre-pregnancy diabetes mellitus is called pregestational diabetes mellitus. Clinically, the other type of diabetes is gestational diabetes mellitus (GDM), which occurs only during pregnancy due to normal glucose metabolism or potential impaired glucose tolerance before pregnancy. More than 80% of pregnant women with diabetes are GDM, and less than 20% are pregestational diabetes mellitus (1, 2).

There are many factors leading to GDM, mainly abnormal glycolipid metabolism (3), which will lead to insulin resistance (4), leading to diabetes. With the deepening of the research, GDM can induce changes in the levels of various factors in the body and act on islet cells to damage insulin, resulting in insulin resistance (5).

Insulin resistance refers to various reasons that reduce the efficiency of insulin in promoting glucose uptake and utilization. The compensatory excessive secretion of insulin in the body produces hyperinsulinemic glycemia to maintain the stability of blood sugar, which is an abnormal pathophysiological state and a common sign of many diseases, especially endocrine and metabolic diseases (6, 7). Insulin resistance easily leads to metabolic syndrome and type II diabetes. GDM can also induce insulin resistance. Our study suspects whether the levels of some factors in serum has an effect on insulin resistance.

Adiponectin and glycated albumin (GA), are selected as the research subjects. Adiponectin is an insulin-sensitizing hormone that can improve insulin resistance and arteriosclerosis in mice (8). Adiponectin level can predict the development of type II diabetes and coronary heart disease. It shows the potential of anti-diabetes, anti-atherosclerosis (9) and inflammation (10) in clinical trials. Glycosylated albumin is a high molecular ketoamine structure formed by the nonenzymatic glycosylation reaction between glucose in the body and the N-terminal of serum protein, 90% of which is combined with lysine at 189th position in the serum protein chain to form the structure of high molecular ketamine, collectively referred to glycated serum protein, of which more than 90% is glycated serum albumin, so GA can reflect the overall level of glycated serum protein (11). GA is suitable for observing the short-term and medium-term changes of blood sugar. Adiponectin and GA are selected as research factors. Moreover, adiponectin and GA secretion abnormalities are closely related to insulin resistance (12).

We investigated the relationship between adiponectin, GA and GDM insulin resistance to provide basis for screening of early GDM.

## Materials and Methods

### General information

Overall, 137 pregnant women with 23–28 gestational weeks examined in Jinan City People's Hospital, Laiwu District, China from Jan 2015 to Jun 2018 were selected. Among them, 71 pregnant women with gestational diabetes mellitus were examined as diabetes group, 66 normal pregnant women as normal pregnant women group. In addition, 58 normal non-pregnant women of childbearing age examined in our hospital during the same period were selected as a control group. The serum adiponectin and GA levels of the three groups were compared, and the relationship between serum adiponectin, GA levels and insulin resistance was analyzed. There was no significant difference among the selected pregnant women.

All subjects and their families agreed to participate in the experiment and signed the informed consent forms, and this study was approved by the Ethics Committee of our hospital (Table 1).

**Table 1:** General information

***Factor***	***GDM group(n=71)***	***Normal pregnant women group (n=66)***	***Control group(n=58)***	***F***	***P***
Age(yr)	26.14±2.21	26.47±2.24	27.07±3.51	1.958	0.1439
Gestational weeks	34.5±2.3	34.8±2.5	-	1.181	0.4931
Gravidity	1.28±0.39	1.25±0.45	-	1.331	0.2405
Pre-pregnancy BMI	23.5±2.14	23.7±2.33	23.3±2.06	0.5188	0.5960
Passive smoking				0.001	0.9995
Yes	43(60.56)	40 (60.61)	35(60.34)		
No	28(39.43)	26(39.39)	23(39.66)		
Education degree				2.782	0.5949
≤ Middle school	11(15.49)	16(24.24)	15(25.86)		
High school	35(49.30)	27(40.91)	23(39.66)		
University	25(35.21)	23(34.85)	20(34.48)		
Menstrual cycle disorder				7.509	0.0234
Yes	46(64.79)	39(59.09)	24(41.38)		
No	25(35.21)	27(40.91)	34(58.63)		
Family history of diabetes				0.1053	0.9487
Yes	21(29.58)	18(27.27)	16(27.59)		
No	50(70.45)	48(72.73)	42(72.41)		

### Inclusion criteria

GDM group and normal pregnancy group were singleton pregnancy. The diagnosis was in accordance with clinical diagnostic criteria for diabetes mellitus (13). Fasting blood glucose (≥ 7 mmol/L) of patients was detected by glucose tolerance test twice or more.

### Exclusion criteria

Patients with diabetes before pregnancy; Patients with history of abortion, premature delivery and fetal death; Patients with abnormal glucose tolerance and insulin resistance; Patients with liver and kidney dysfunction or serious immune diseases.

### Methods and Observation Standards

All pregnant women were fasted for 12∼14 h during sugar screening, and fasting venous blood was taken in the morning of the next day. The blood was put into a centrifuge and centrifuged for 7–10 min at 3500 rpm. Serum was collected and stored at −80 °C for testing. FPG levels in each group were measured by glucose oxidase method (14). Fasting insulin (FINS) level was measured by immunofluorescence (15). Insulin resistance index (16) (HOMA-IR) was evaluated by steady-state model. HOMA-IR= FINS* FPG/22.5. Serum GA and adiponectin levels were determined by ELISA (17). GA kit was purchased from Shanghai Enzyme-linked Biotechnology Co., Ltd. with the product number ml024082; Adiponectin kit was purchased from Shanghai Enzyme-linked Biotechnology Co., Ltd. with the product number ml061301.

### Statistical methods

SPSS 18.0 software (Biz Insight (Beijing) Information Technology Co., Ltd.) was used to carry out statistical analysis on the data. GraphPad Prism 6 was used to visualize all the pictures in this experiment, and the counting data were expressed by rate (%). Chi-square test was used for the comparison, Pearson correlation analysis for relations among variables, *t*-test for normal distribution, rank sum test for non-normal distribution, and the measurement data were expressed by the mean± standard deviation. *t*-test was used for analysis between the two groups, variance analysis was used for comparison among multiple groups. Receiver operating characteristic (ROC) curve was used for analysis of the diagnostic value of adiponectin and GA in GDM pregnant women. The *P* value less than 0.050 was regarded as statistical significance.

## Results

### Comparison of OGTT results among three groups

**Fig. 1:**
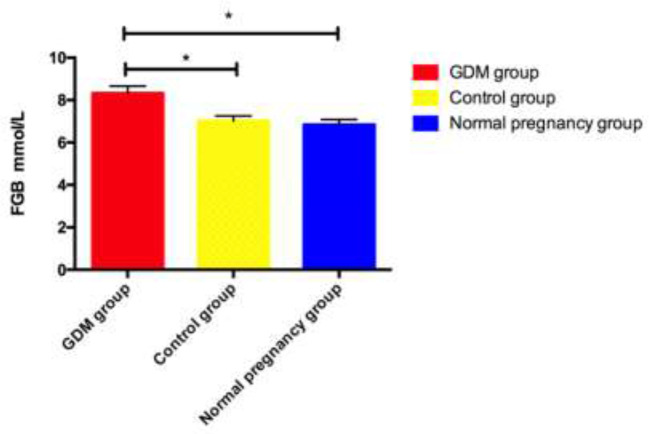
Comparison of OGTT results among three groups * indicates that *P*<0.05

The level of FBG in the GDM group was significantly higher than that of the normal pregnancy group and the control group (*P*=0.031, *P*=0.027), and was slightly higher in the normal pregnancy group than in the control group, the difference was not statistically significant (Fig. 1).

### Levels of adiponectin and GA in Serum of Three Groups

**Fig. 2:**
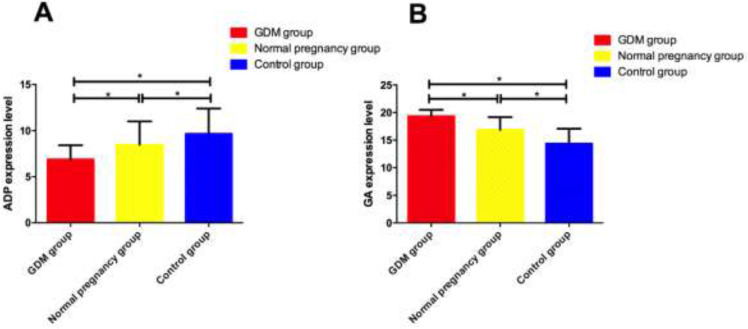
Comparison of adiponectin and GA levels in three groups **A:** Comparison of adiponectin levels in three groups. **B:** Comparison of GA levels in three groups. * indicates that *P*<0.05

The level of adiponectin in serum of GDM group was significantly lower than that of normal pregnancy group and control group, and there were statistical differences between the control group and the normal pregnancy group (*P*<0.05). The level of GA in serum of GDM group was significantly higher than that of normal pregnancy group and control group, and there were statistical differences between the control group and the normal pregnancy group (*P*<0.05) (Fig. 2).

### Comparison of FPG, FINS and HOMA-IR levels

**Fig. 3:**
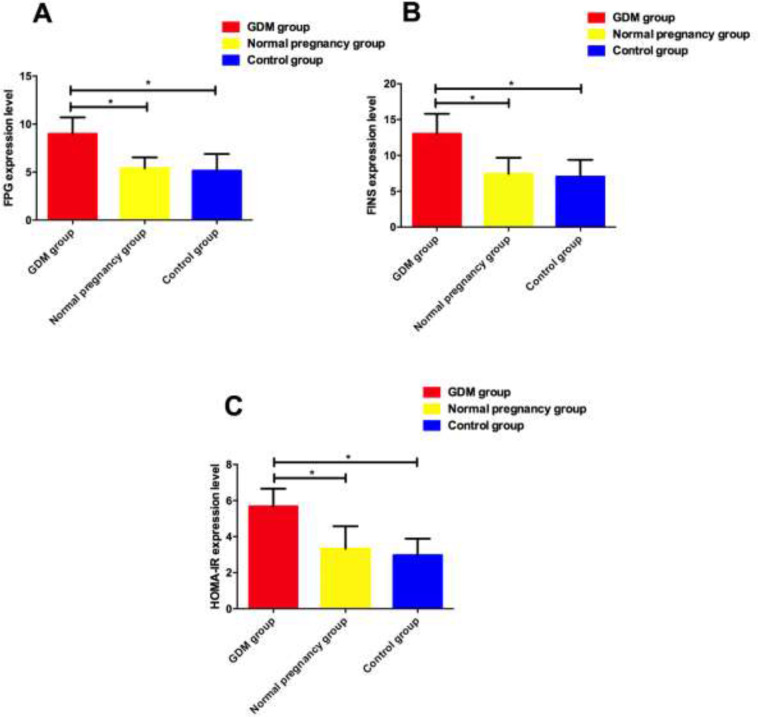
Comparison of FPG, FINS and HOMA-IR levels of three groups **A:** Comparison of FPG levels of three groups. **B:** Comparison of FINS levels of three groups. **C:** Comparison of HOMA-IR levels of three groups. * indicates that *P*<0.05

FPG, FINS, HOMA-IR in GDM group were higher than those in normal pregnancy group (*P*<0.001), while FPG, FINS, HOMA-IR in normal pregnancy group were higher than those in normal non-pregnancy group (*P*>0.05) (Fig. 3).

### Correlation analysis

**Fig. 4:**
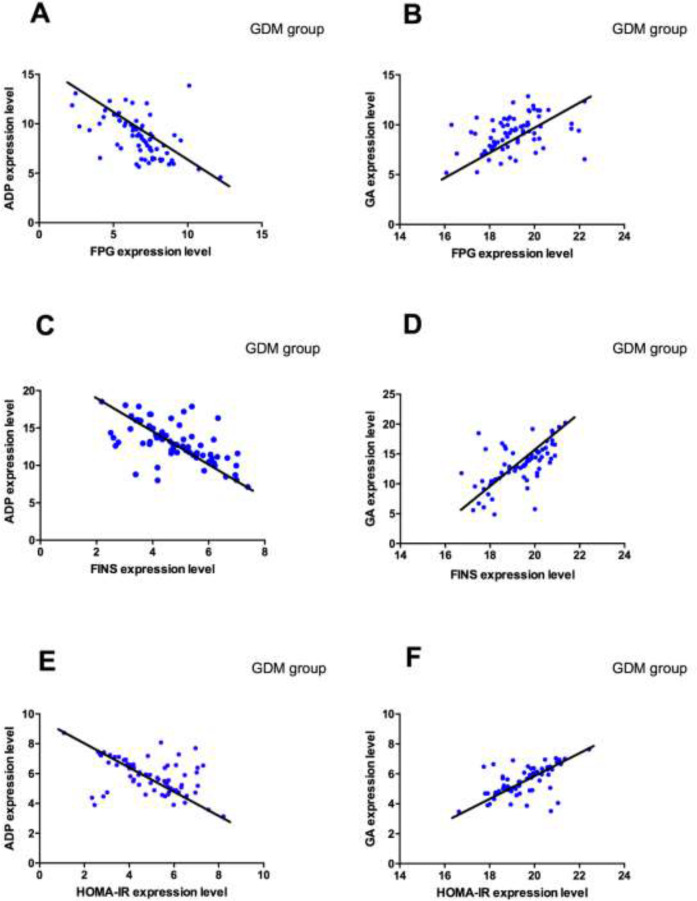
Correlation analysis of adiponectin, GA and FPG, FINS, HOMA-IR in GDM group. **A:** Correlation analysis of adiponectin and FPG in GDM group; **B:** Correlation analysis of GA and FPG in GDM group; **C:** Correlation analysis of adiponectin and FINS in GDM group; **D:** Correlation analysis of GA and FINS in GDM group; **E:** Correlation analysis of adiponectin and HOMA-IR in GDM group; **F:** Correlation analysis of GA and HOMA-IR in GDM group. *P*<0.05 indicates that there was a correlation

Pearson correlation analysis showed that adiponectin was negatively correlated with FPG, FINS and HOMA-IR (*P*<0.001), while GA was positively correlated with FPG, FINS and HOMA-IR (*P*<0.001) (Fig. 4).

### ROC curve analysis

**Fig. 5:**
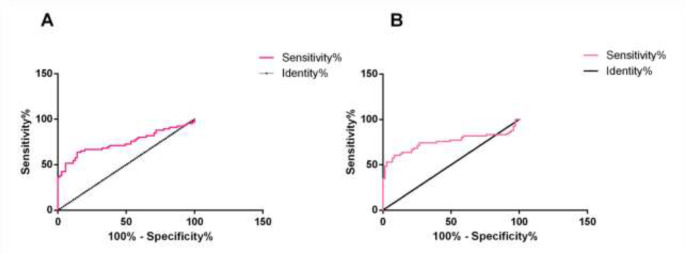
ROC curve analysis of adiponectin and GA in diagnosis of DGM **A** ROC curve analysis of adiponectin in diagnosis of GDM; **B:** ROC curve analysis of GA in diagnosis of GDM

ROC curve analysis of adiponectin and GA showed that the sensitivity of GA was 91.55%, specificity was 60.61%, AUC was 0.7505, and the sensitivity of adiponectin was 85.92%, specificity was 63.64%, AUC was 0.7420. The area under the ROC curve (AUC) of GA was larger than that of adiponectin, the sensitivity of GA was higher than that of adiponectin, and the specificity was lower than that of adiponectin (Fig. 5).

## Discussion

Gestational diabetes mellitus has relatively high risks and adverse effects on pregnant women and fetuses. Research on hyperglycemia and adverse pregnancy outcomes (18) (HAPO research) showed that abnormal blood glucose significantly increased the incidence of maternal and infant adverse outcomes such as neonatal hypoglycemia, which seriously endangered maternal and infant health. However, the pregnancy process was a physiological insulin resistance state, but not all pregnant women had gestational diabetes, and some returned to normal after delivery (19). In order to further illustrate that insulin resistance is one of the pathophysiological bases of GDM, GDM had more severe IR than normal pregnancy (20). Clinically, the main indexes for diagnosis of insulin resistance were FPG level and FINS level. The insulin (HOMA-IR) was evaluated. Then FPG, FINS and HOMA-IR levels of three groups of patients were compared. The results were consistent with another research (20), and IR level in GDM group significantly increased. Therefore, it was confirmed that insulin resistance was one of the physiological and pathological bases of GDM.

In another study, adiponectin level was positively correlated with insulin sensitivity (21), suggesting that adiponectin reduction was an independent risk factor for IR and GDM. By Pearson correlation factor analysis, the results of this study showed that the adiponectin level in GDM patients decreased, and was negatively correlated with FPG, FINS and HOMA-IR levels (*P*<0.05). The adiponectin level in GDM patients was significantly lower than that in normal pregnant women (22). GA level in GDM patients was higher than that in normal pregnant women (23), consistent with the results of this study. It showed that the occurrence of GDM was related to adiponectin and GA, which had an effect on the pathogenesis of GDM.

The results of this study showed that the GA level of blood glucose in GDM group was significantly higher than that in normal pregnancy group and normal non-pregnancy group, which indicated that the high-risk group of GDM can be effectively screened by measuring GA level. Consistent with another study (24), GA was a product of binding albumin lysine stump to glucose carbonyl. The level of GA in serum was related to the half-life of albumin and blood glucose concentration (25). Under normal circumstances, GA concentration in the body is relatively stable, and is positively related with the blood sugar in a period of time, which can reflect the blood sugar stability in 2–3 weeks. Therefore, the blood sugar metabolism of GDM pregnant women can be observed through GA level, which is helpful for blood sugar monitoring of GDM pregnant women.

Adiponectin is an active polypeptide secreted by white adipocytes. Its main physiological function in the body is to maintain blood sugar homeostasis and regulate lipid metabolism. Adiponectin also has the functions of anti-inflammation, anti-atherosclerosis and insulin sensitization. Adiponectin level of pregnant women is mainly a risk factor for diabetes, which can strengthen fatty acid oxidation function and reduce body lipid concentration (26). The adiponectin level of pregnant women was negatively correlated with insulin resistance index (26). When serum adiponectin drops by 1%, the risk of GDM will increase 4.25 times (27). Moreover, animal experiments proved that adiponectin can achieve hypoglycemic effect by improving IR function of mice (28, 29). The mechanism of adiponectin was to specifically bind G protein-coupled receptors on skeletal muscle and liver cell membranes, and then regulate fatty acid oxidation and glycometabolism (30). Park et al (31) suggested that adiponectin was negatively correlated with FINS. The results of this study were basically consistent with the above results, and adiponectin was significantly negatively correlated with HOMA-IR, FINS, pre-pregnancy BMI, pre-pregnancy waist circumference, etc. Adiponectin is related to insulin resistance and has the function of resistance to insulin (31). Its mechanism is that adiponectin can directly stimulate the phosphorylation of 5-AMP activated protein kinase in skeletal muscle and liver, leading to acetylcoa carboxylase phosphorylation, promoting the oxidation of fatty acids and the increase of glucose uptake (32). Adiponectin can inhibit liver gluconeogenesis, improve insulin resistance and reduce triglyceride content by increasing the oxidation of fatty acids in skeletal muscle, thereby improving insulin resistance.

The results of this study showed that the serum adiponectin level of GDM patients was significantly increased, showing a positive correlation, and the level was significantly higher than that of normal pregnant group and normal non-pregnant group, thus indicating that the decrease of adiponectin level may be closely related to the occurrence and progression of GDM.

The ROC curve showed that the sensitivity of GA was 91.55%, specificity was 60.61%, AUC was 0.7505, the sensitivity of adiponectin was 85.92%, specificity was 63.64%, AUC was 0.7420. The main diagnostic method of GDM in clinic is to carry out glucose tolerance test for screening, and glucose tolerance test is easy to be affected by various factors, such as diet, drugs, emotions, etc., which easily lead to false positive results. However, the accuracy of DGM diagnosis can be improved by measuring adiponectin and GA levels in serum. In addition, it should be noted that besides IR, there are many factors involved in the pathogenesis of GDM, and adiponectin and GA are only related to IR, so the detection of adiponectin and GA can only be used as a complementary diagnostic method for GDM, and cannot replace the traditional glucose tolerance test. This experiment failed to further explore more pathogenesis of GDM, so we hope that the majority of scholars can carry out further in-depth research on this. We will also take further researches in this direction.

## Conclusion

Combined with the results, abnormal adiponectin and GA levels are closely related to islet resistance in GDM patients, and detection of adiponectin and GA serum levels can provide help for early diagnosis of GDM.

## Ethical considerations

Ethical issues (Including plagiarism, informed consent, misconduct, data fabrication and/or falsification, double publication and/or submission, redundancy, etc.) have been completely observed by the authors.
